# Intimate partner violence and prevention of mother to child transmission of HIV: Evidence from Kinshasa, Democratic Republic of Congo

**DOI:** 10.1371/journal.pone.0203471

**Published:** 2018-08-30

**Authors:** Margaret W. Gichane, Kathryn E. Moracco, Harsha Thirumurthy, Emile W. Okitolonda, Frieda Behets, Marcel Yotebieng

**Affiliations:** 1 The University of North Carolina at Chapel Hill, Department of Health Behavior, Chapel Hill, NC, United States of America; 2 University of Pennsylvania Pearlman School of Medicine, Department of Medical Ethics and Health Policy, Philadelphia, PA, United States of America; 3 The University of Kinshasa, School of Public Health, Kinshasa, Democratic Republic of Congo; 4 The University of North Carolina at Chapel Hill, Department of Epidemiology, Chapel Hill, NC, United States of America; 5 The University of North Carolina at Chapel Hill, Department of Social Medicine, Chapel Hill, NC, United States of America; 6 The Ohio State University, College of Public Health, Division of Epidemiology, Columbus, OH, United States of America; Asociacion Civil Impacta Salud y Educacion, PERU

## Abstract

Intimate partner violence (IPV) is a risk factor for non-adherence to HIV treatment for women, however the evidence on the impact of IPV on uptake of the prevention of mother to child transmission of HIV (PMTCT) cascade is inconclusive. We examined data from 433 HIV positive pregnant women in Kinshasa, Democratic Republic of Congo, enrolled between April 2013 and August 2014 and followed-up through 6 weeks postpartum. Participants were asked about their IPV experiences in a face-to-face interview at enrollment. Measures of PMTCT cascade included: uptake of clinical appointments and services, viral suppression, and adherence to antiretrovirals (ARV). Approximately half of the sample (51%) had experienced some form of IPV; 35% had experienced emotional abuse, 29% physical abuse, and 19% sexual abuse. There were no statistically significant associations between experiencing any form of IPV and uptake of clinical appointments and services (Adjusted Prevalence Ratio [aPR] = 1.02; 95% [CI]: 0.89–1.17), viral load suppression (aPR = 1.07, 95% CI:0.96–1.19) and ARV adherence (aPR = 1.01, 95% CI: 0.87–1.18). Findings from this study indicate that, among HIV-infected pregnant women enrolled in PMTCT care, experiencing IPV does not reduce adherence to clinic visits and services, adherence to ARV. The high prevalence of IPV in this population suggests that IPV screening and intervention should be included as part of standard care for PMTCT.

## Introduction

Prevention of mother to child transmission of HIV (PMTCT) is an essential component of global strategies to reduce pediatric HIV infection [1]. The World Health Organization (WHO) guidelines for PMTCT outline a “cascade” of behaviors and services including: antenatal care appointments, HIV counseling and testing, use of antiretroviral therapy (ARV), delivery at a health facility, safe infant feeding, and infant HIV testing [2]. Expansion of PMTCT coverage reduced incidence of pediatric HIV infection by 60% in Sub-Saharan Africa (SSA) from 2008–2015 annually [3]. However, uptake of services along the PMTCT cascade remains sub-optimal. In the Democratic Republic of Congo (DRC), only 67% of pregnant women living with HIV received ARV for PMTCT in 2015 [4].

Understanding factors that may impede the uptake of PMTCT services are of critical importance to curb pediatric HIV incidence. Intimate partner violence (IPV) is a significant global health issue which has been found to decrease adherence to HIV treatment and engagement in care among HIV infected women [5]. A systematic review of IPV prevalence among pregnant women in Africa found that 2–57% of women experience some form of IPV during pregnancy. Emotional violence was most commonly reported (41–49%), followed by physical (22.5–40%), and sexual violence (2.7–26.5%) [6]. Despite the evidence documenting IPV among pregnant women living with HIV [6–10], its impact on PMTCT service uptake is not well understood.

Studies assessing the effect of IPV on PMTCT uptake and adherence have yielded varying findings depending on which components of the PMTCT cascade are assessed. A retrospective study of ARV adherence among 320 postpartum women in Lusaka, Zambia found that women who had experienced IPV were less likely to: adhere to ARV during and after pregnancy, and give their infant nevirapine after birth [11]. These findings are supported by qualitative studies that cited IPV as a barrier to ARV adherence and clinic attendance for pregnant women living with HIV [9, 10, 12, 13].

In contrast, other studies of IPV and PMTCT indicate that IPV does not inhibit certain aspects of PMTCT service uptake [14, 15]. A study conducted with 457 women in Uganda found that IPV was not associated with antenatal attendance, uptake of HIV testing and counseling, and delivery at a health facility [14]. HIV status was not collected, and thus findings may not be relevant to women living with HIV. Another study conducted in Kenya that included women living with HIV found that IPV did not inhibit HIV positive women from giving a single dose of nevirapine to their infants at birth [15].

There are different mechanisms through which IPV may inhibit women’s ability to adhere to PMTCT. Fear of physical or emotional violence due to HIV status disclosure may make some women hide or discontinue use of ARV, or miss medical appointments [9, 10, 16, 17]. IPV may also cause depression which is a known risk factor for ARV non-adherence [18] and poor HIV care engagement [19]. The conflicting results across studies indicates that a history of IPV may impact some components of the PMTCT cascade, however there are no studies to date that take a comprehensive analysis of both PMTCT service uptake and maternal HIV outcomes. The aim of the present study was to explore whether emotional, physical, or sexual violence differentially impact uptake of PMTCT clinical appointments and services, viral suppression, and ARV adherence among HIV infected pregnant women. We hypothesize that women who experience any forms of violence will be less likely to adhere to the PMTCT cascade.

## Methods

### Study design and procedures

The data reported here are from a randomized controlled trial of a conditional cash transfer (CCT) study that was conducted in Kinshasa, Democratic Republic of Congo (Trial registration: NCT01838005) [20]. From April 2013 to August 2014, 433 newly-diagnosed HIV-infected women, ≤32 weeks pregnant were recruited as they registered for antenatal care at 89 clinics. Women were randomly assigned to the control or intervention using a computer generated randomization technique. Clinical staff were masked to women’s intervention condition. Control group received the standard of care which consisted of: free ARV, HIV testing and counseling, CD4 testing, HIV prevention and care for HIV-exposed infants, and linkage to HIV clinics by HIV positive volunteers. Women randomized to the intervention received the standard of care and small increasing cash payments for each activity they completed in the PMTCT cascade with a reset option to zero if a step was missed. Women were interviewed and provided written informed consent upon enrollment by trained research staff in Lingala or French. The study was approved by the Institutional Review Board of the Ohio State University (Colombus, OH, USA) and the Ethical Committee of the Kinshasa School of Public Health (Kinshasa, Democratic Republic of the Congo).Study details and primary results have been described elsewhere [20, 21].

### Measures

The exposure variable of interest was baseline report of lifetime IPV. Participants were screened for IPV upon enrollment using a screening tool developed for use in antenatal care clinics in Kinshasa as a component of voluntary HIV counseling and testing during the first antenatal visit. The tool contained questions assessing lifetime and 12-month emotional, physical abuse by a current or former intimate partner, and sexual abuse from any perpetrator, as these experiences were deemed most likely to increase a woman’s risk of HIV infection. Emotional violence was assessed using the following item, “Have you ever been insulted, humiliated, or made to feel afraid by an intimate partner?,” which was adapted from items developed by the international WHO Multi-country Study on Women's Health and Domestic Violence against Women Study Team [22, 23]. For physical violence, we used the item “Have you ever been hit, punched, kicked, slapped, choked, or otherwise physically hurt by an intimate partner?”, which was adapted from a similar item from the Abuse Assessment Scale (AAS) [24]. Finally, sexual violence was assessed with, “Have you ever been forced to have sex or do something sexual you didn’t want to do?,” which was adapted from the Youth Behavior Risk Survey item measuring sexual violence victimization [25]. Response options were “yes” or “no.” If participants answered yes, they were asked who committed the violence. Response options included, “husband/ex-husband,” “partner/ex-partner,” “other,” “don’t know,” and “refused to answer.” Women were also asked whether violence occurred within the last 12 months and the most recent time violence occurred. For each form of IPV (emotional, physical, sexual) and a composite of any type of IPV, women were categorized as having experienced IPV if they answered “yes” to any violence question.

The outcome measures assessed in the study were uptake of PMTCT services, viral suppression, and ARV adherence measured at six weeks post-partum. Uptake of PMTCT services was a composite measure of the completion of several clinical steps of the PMTCT cascade. A participant was considered adherent if she completed all of the following steps: 1) attended all scheduled clinic visits from randomization through six weeks postpartum; 2) initiated ARV and HIV care; 3) gave birth in a study clinic; 4) and accepted all proposed clinical services including provision of blood samples for CD4 count and dried blood spot sample for DNA PCR testing at six weeks. Infant nevirapine use at birth and six weeks post-partum, and infant DNA PCR testing at six weeks were not included as a measure of adherence to PMTCT services due to resource limitations in the study clinics.

Viral suppression was assessed as viral load count obtained via a dried blood spot (5 spots of 50 mL of whole blood) at follow-up clinic appointments. Participants were considered virally suppressed if they had an undetectable viral load at six weeks postpartum. ARV adherence was determined by pill counts. Clinic staff calculated the number of returned pills [zidovudine (AZT) or ART] divided by the number of days since the pills were distributed. Participants with 100% adherence at all visits were considered adherent, whereas those with less than 100% adherence were considered non-adherent.

### Data analysis

Descriptive statistics of IPV history and demographics were calculated. We created dummy variables for any experience of IPV and type of IPV (emotional, physical, sexual). We fit generalized estimating equations (GEE) with a log link and a binomial distribution to adjust for clustering at the clinic level (89 clinics, average cluster size = 5.2). We estimated unadjusted and adjusted prevalence ratios (APR) and 95% confidence intervals (CIs) for each study outcome. Analyses were conducted using SAS version 9.4 (Cary, NC). All statistical tests were two-tailed, using an alpha of 0.05. All models controlled for demographic variables including participant education level, marital status, age, and intervention group. Analyses for ARV adherence and viral suppression also controlled for baseline viral suppression and CD4 count.

## Results

### Participants

Participant characteristics are reported in Table 1. The mean age of women in the sample was 29.58 (SD = 6.21) and ages ranged from 16–43 years. The majority of women had a secondary education or higher (85%, 368/433) and were married or cohabiting with a primary partner (82%, 358/433). For those with available viral load data at baseline, 52% (169/326), were undetectable.

**Table 1:** Characteristics of participants (n = 433).

	N (%)
**Age in years (SD)**	29.58 (6.21)
**Gestational age in weeks (SD)**	25.11 (4.64)
**Primiparus**	
Yes	56 (12.39)
No	377 (87.07)
**Maternal education level**	
Primary	65 (15.01)
Secondary	319 (73.67)
Higher than secondary	49 (11.32)
**Marital Status**	
Married/cohabiting	358 (82.87)
Divorced/separated/widow/never married	74 (17.13)
**Transport to clinic**	
Walk	245 (56.58)
Other	188 (43.42)
**LTFU**	
No	366 (84.53)
Yes	67 (15.47)
**Uptake of PMTCT service**	
No	171 (39.49)
Yes	262 (60.51)
**Baseline viral load**	
Undetectable	169 (51.84)
Detectable	157 (48.16)

Over half (51%, 221/433) of the sample had experienced at least one form of IPV in their lifetime (Table 2). Emotional violence was the most commonly reported (35%, 152/433), followed by physical (29%, 124/433), and sexual violence (19%, 82/433). A smaller proportion of participants had experienced each violence type within the last 12 months. Still, nearly a third (32%, 138/433) had been abused by an intimate partner over the last year.

**Table 2:** Participant history of intimate partner violence (IPV) (n = 433).

	All N (%)	Within the last 12 months
**Any IPV**		
No	212 (48.96)	295 (68.13)
Yes	221 (51.04)	138 (31.87)
**Emotional Violence**		
No	281 (64.90)	332 (76.66)
Yes	152 (35.10)	101 (23.33)
**Physical Violence**		
No	309 (71.36)	359 (82.91)
Yes	124 (28.64)	74 (17.09)
**Sexual Violence**		
No	351 (81.06)	366 (84.53)
Yes	82 (18.94)	67 (15.47)

### PMTCT service uptake

At six weeks postpartum 61% (262/433) of the sample reported full uptake of all PMTCT appointments and clinical services. There was no statistically significant effect of IPV experience (aPR: 1.02, 95% CI: 0.89–1.17) on PMTCT service uptake. Non-significant effects were also found when emotional, physical, and sexual abuse were analyzed separately (Table 3).

**Table 3:** Association between IPV history and Adherence to PMTCT (n = 433).

Adherence to PMTCT				
	Yes	No	PR (95% CI)	aPR (95% CI)
**Any IPV**				
No	126 (59.43)	86 (40.57)	Ref	Ref
Yes	136 (61.54)	85 (38.46)	1.03 (0.89–1.20)	1.02 (0.89–1.17)
**Emotional Violence**				
No	167 (59.43)	114 (40.57)	Ref	Ref
Yes	95 (62.50)	57 (37.50)	1.05 (0.89–1.24)	1.07 (0.91–1.25)
**Physical Violence**				
No	187 (60.52)	122 (39.48)	Ref	Ref
Yes	75 (60.48)	49 (39.52)	1.00 (0.85–1.17)	1.00 (0.86–1.18)
**Sexual Violence**				
No	207 (58.97)	144 (41.03)	Ref	Ref
Yes	55 (67.07)	27 (32.93)	1.14 (0.95–1.35)	1.07 (0.89–1.28)

Ref = referent group

Adjusted analyses control for: intervention group age, marital status, and maternal education.

### Viral suppression

Data on viral suppression were available for 326 participants. Approximately 68% (221/326) had an undetectable viral load at six weeks postpartum. Similar to findings for PMTCT service uptake, IPV experience was not significantly associated with viral suppression (aPR: 1.07, 95% CI: 0.96–1.19). Likewise, effects were non-significant for emotional, physical, and sexual abuse (Table 4).

**Table 4:** Association between IPV history and viral suppression at 6 weeks follow-up (n = 326).

Viral Suppression				
	Yes	No	PR (95% CI)	aPR (95% CI)
**Any IPV**				
No	108 (66.7)	54 (33.3)	Ref	Ref
Yes	113 (68.9)	51 (31.1)	1.03 (0.89–1.19)	1.07 (0.96–1.19)
**Emotional Violence**				
No	144 (68.2)	67 (31.8)	Ref	Ref
Yes	77 (67.0)	38 (33.0)	0.98 (0.84–1.14)	1.05 (0.96–1.14)
**Physical Violence**				
No	157 (66.8)	78 (33.2)	Ref	Ref
Yes	64 (70.3)	27 (29.7)	1.05 (0.90–1.23)	1.07 (0.95–1.20)
**Sexual Violence**				
No	180 (68.2)	84 (31.8)	Ref	Ref
Yes	41 (66.1)	21 (33.9)	0.97 (0.80–1.17)	1.07 (0.90–1.28)

Adjusted analyses control for: intervention group, age, marital status, maternal education, baseline viral suppression, and baseline CD4 count.

### ARV adherence

Approximately 69% (205/297) of participants with available pill count data were 100% adherent at all visits up to six weeks postpartum. There were no significant differences in ARV adherence among those who had experience any violence versus those who had not (aPR: 1.01, 95% CI: 0.87–1.18). Effects remained non-significant when violence types were analyzed separately (Table 5).

**Table 5:** Association between IPV history and adherence to ART (n = 297).

	Adhered to ART			
	Yes	No	PR (95% CI)	aPR (95% CI)
**Any IPV**				
No	96 (66.2)	49 (33.8)	Ref	Ref
Yes	109 (71.7)	43 (29.3)	1.08 (0.93–1.26)	1.01 (0.87–1.18)
**Emotional Violence**				
No	126 (66.0)	65 (34.0)	Ref	Ref
Yes	79 (74.5)	27 (25.5)	1.13 (0.98–1.31)	1.06 (0.90–1.24)
**Physical Violence**				
No	146 (69.2)	65 (30.8)	Ref	Ref
Yes	59 (68.6)	27 (31.4)	0.99 (0.85–1.16)	1.00 (0.86–1.16)
**Sexual Violence**				
No	162 (67.8)	77 (32.2)	Ref	Ref
Yes	43 (74.1)	15 (25.9)	1.09 (0.92–1.29)	1.06 (0.90–1.25)

Adjusted analyses control for: intervention group, age, marital status, maternal education, baseline viral suppression, and baseline CD4 count.

## Discussion

Contrary to our hypothesis, we did not find any statistically significant effect of IPV history on uptake of PMTCT clinical appointments and services, ARV adherence, and viral suppression. Our study is the first to assess the impact of IPV on maternity care use in a sample of women living with HIV. Specifically we assessed antenatal care attendance, skilled delivery at a study clinic, and acceptance of tests and HIV care as part of PMTCT service uptake. We did not find a significant relationship between history of any form of IPV and components of PMTCT service uptake. Previous literature on the impact of IPV on antenatal care utilization has been inconclusive. A recent study conducted in Rwanda found that there are no significant differences in antenatal use between women who have and have not experienced physical, emotional or sexual IPV [26]. However, other studies found the opposite. History of IPV was associated with low antenatal care attendance and lack of skilled attendant at delivery [27–29]. A plausible explanation for the lack of significant findings is the fact that women were recruited as they registered for antenatal care. This sample thus may have already been motivated to remain in care because they took the initial step of registering at a clinic. Furthermore, the standard of care offered at study clinics included HIV testing and counselling, support in ARV initiation, as well as volunteers to assist with HIV care linkage. These components of care likely provide patients with needed instrumental, informational, and emotional social support [30]and thus may potentially circumvent barriers potentially caused by violence from an intimate partner. Research shows that among women who have experienced IPV, higher social support is associated with reduced risk of poor mental and physical health outcomes [31]. Future studies should explore how and which support systems and services buffer the impact of IPV and maternity care use.

Our findings conflict with previous work by Hampanda [11]which found a statistically significant association between IPV experience and ARV adherence during pregnancy and six weeks postpartum. Women who had experienced IPV were not less likely to adhere to HIV medication or to have a detectable viral load. Additionally, we did not see differences in ARV adherence based on the type of violence women had experienced. These results counter a large body of evidence linking IPV to ARV non-adherence and worsened viral suppression in women [5]. The lack of significant association between IPV and ARV adherence may be attributed to differences in measurement across studies. Both our study and the one by Hampanda [11] used a dichotomous measure of adherence, however our cutoff for ‘adherent’ was 100% whereas theirs was 80%. Furthermore, we looked at the impact of IPV on cumulative adherence through pregnancy and postpartum whereas they assessed the impact of IPV on adherence separately for pregnancy and postpartum. It is possible that a history of IPV may affect ARV adherence differently at various stages of pregnancy and postpartum. Further research on the mechanisms linking IPV to ARV adherence is needed.

One potential explanation for the lack of a significant relationship between IPV and viral suppression or ARV adherence is that during pregnancy, women show a high level of motivation and self- efficacy in taking their medication in order to protect their infants from HIV. A systematic review of ARV adherence among pregnant and postpartum women found that approximately 73.5% (95% CI: 71·5–79·7%) of women are adherent during pregnancy and 53% (95% CI: 32.8%-72·7%) are adherent during the postpartum period [32]. We found that 68% of women remained 100% adherent from pregnancy through six weeks postpartum. This high level of sustained adherence is promising as it reduces the probability of mothers transmitting HIV to their infants via breastfeeding in the postpartum period.

Our findings are consistent with previous studies that show pregnant women living with HIV experience high levels of IPV [6–8, 11]. The life time prevalence of IPV found in the sample was 51% which is on the high end of global estimates [6], but lower than the national average for DRC, 68.2% [33]. In addition, nearly a third had experienced IPV within the last year, which suggests that some women may have been abused during their pregnancy and in the post-partum period. IPV during pregnancy is of particular concern as it is associated with poor pregnancy outcomes including: pregnancy loss, preterm labor, pregnancy complications, maternal depression, low birth weight infant, physical injuries, stress, and maternal mortality [34]. Further, among post-partum women living with HIV, IPV during is also associated with infant developmental delays [35]. The high prevalence of IPV prior to and during pregnancy indicates the critical need for screening and IPV prevention services for pregnant women living with HIV.

The main strengths of the study were in the measures, study design, and sampling frame. Our study was the first to assess various components of the PMTCT cascade including: antenatal attendance, ARV adherence, and viral suppression in a sample of pregnant women living with HIV. We used viral load tests which is the recommended method of measuring ARV adherence according to the WHO [2]. In addition, our data were longitudinal nature so we can establish temporal order of IPV and adherence. Finally, participants were recruited from 89 different clinics in Kinshasa which represented both high-volume, low-volume, public and private clinics. Findings may be applicable to a variety of clinical settings in low-income countries.

Several limitations must be considered in interpretation of our results. These data represent a secondary analysis of intervention data. As such, the original study was not powered to detect significant differences in PMTCT cascade outcomes based on IPV experience. IPV experience was also self-reported which may be subject to recall bias. Additionally, our sample was highly educated with a secondary completion rate much higher than the general population (85% sample vs. 48% general population) [36], which limits generalizability of the findings. Finally, we did not include measures of infant nevirapine uptake at birth and six weeks postpartum as part of ARV adherence. There is a possibility that these two components of PMTCT may be affected by IPV, especially if the mother is currently experiencing IPV within her relationship. However, given the consistency of our findings for all the other PMTCT outcomes we assessed, we do not believe it is likely that infant nevirapine use would have yielded different results.

## Conclusions

We did not find any statistically significant associations between IPV history and uptake of the PMTCT services, ARV adherence, and viral suppression. However, IPV was highly common in a sample of pregnant women living with HIV in Kinshasa, DRC. Identifying pregnant women who have experienced IPV is of critical importance because IPV victimization is associated with numerous poor health outcomes including: contraction of sexually transmitted infections, induced abortion, PTSD, depression, alcohol use disorders, and injury [37]. PMTCT interventions present an opportune time to screen for violence because women meet with healthcare providers privately to discuss their HIV test results and then several times during their pregnancy and postpartum period, which allows for time to build rapport and develop strategies to identify and address IPV. Healthcare providers should be trained on how to effectively screen women for IPV during PMTCT antenatal appointments. This study contributes novel evidence that the impact of IPV on adherence may be lessened during pregnancy and postpartum. Our findings illustrate the need to further study the mechanisms linking IPV to non-adherence.

## References

[1] UNAIDS. Countdown to Zero: Global plan towards the elimination of new HIV infections among children by 2015 and keeping their mothers alive. Geneva, Switzerland: 2011.

[2] WHO. Consolidated guidelines on diagnosis, treatment and care for key populations HIV prevention: 2016 update. Geneva, Switzerland: 2016.

[3] UNAIDS. On the fast-track to an AIDS free generation. Geneva, Switzerland: 2016.

[4] UNAIDS. Democratic Republic of the Congo: 2015 Country factsheets http://aidsinfo.unaids.org/2015 [cited 2017 September 13].

[5] Hatcher AM, Smout EM, Turan JM, Christofides N, Stockl H. Intimate partner violence and engagement in HIV care and treatment among women: a systematic review and meta-analysis. AIDS. 2015;29(16):2183–94. 10.1097/QAD.0000000000000842 . 26353027

[6] Shamu S, Abrahams N, Temmerman M, Musekiwa A, Zarowsky C. A systematic review of African studies on intimate partner violence against pregnant women: prevalence and risk factors. PLoS One. 2011;6(3):e17591 10.1371/journal.pone.0017591 ; PubMed Central PMCID: PMCPMC3050907. 21408120 PMC3050907

[7] Bernstein M, Phillips T, Zerbe A, McIntyre JA, Brittain K, Petro G, et al Intimate partner violence experienced by HIV-infected pregnant women in South Africa: a cross-sectional study. BMJ Open. 2016;6(8):e011999 10.1136/bmjopen-2016-011999 ; PubMed Central PMCID: PMCPMC5013465. 27531735 PMC5013465

[8] Ezeanochie MC, Olagbuji BN, Ande AB, Kubeyinje WE, Okonofua FE. Prevalence and correlates of intimate partner violence against HIV-seropositive pregnant women in a Nigerian population. Acta Obstet Gynecol Scand. 2011;90(5):535–9. 10.1111/j.1600-0412.2011.01083.x . 21306341

[9] Hatcher AM, Stockl H, Christofides N, Woollett N, Pallitto CC, Garcia-Moreno C, et al Mechanisms linking intimate partner violence and prevention of mother-to-child transmission of HIV: A qualitative study in South Africa. Social Science and Medicine. 2016;168:130–9. 10.1016/j.socscimed.2016.09.013 . 27643847

[10] Hatcher AM, Woollett N, Pallitto CC, Mokoatle K, Stockl H, MacPhail C, et al Bidirectional links between HIV and intimate partner violence in pregnancy: implications for prevention of mother-to-child transmission. Journal of the International AIDS Society. 2014;17:19233 10.7448/IAS.17.1.19233 ; PubMed Central PMCID: PMCPMC4220001. 25371218 PMC4220001

[11] Hampanda KM. Intimate partner violence and HIV-positive women's non-adherence to antiretroviral medication for the purpose of prevention of mother-to-child transmission in Lusaka, Zambia. Social Science and Medicine. 2016;153:123–30. 10.1016/j.socscimed.2016.02.011 ; PubMed Central PMCID: PMCPMC4788551. 26896876 PMC4788551

[12] Kosia A, Kakoko D, Semakafu AME, Nyamhanga T, Frumence G. Intimate partner violence and challenges facing women living with HIV/AIDS in accessing antiretroviral treatment at Singida Regional Hospital, central Tanzania. Global Health Action. 2016;9(1):32307 10.3402/gha.v9.32307 27987296 PMC5161793

[13] Mepham S, Zondi Z, Mbuyazi A, Mkhwanazi N, Newell ML. Challenges in PMTCT antiretroviral adherence in northern KwaZulu-Natal, South Africa. AIDS Care. 2011;23(6):741–7. 10.1080/09540121.2010.516341 . 21293987

[14] Karamagi CA, Tumwine JK, Tylleskar T, Heggenhougen K. Intimate partner violence against women in eastern Uganda: implications for HIV prevention. BMC Public Health. 2006;6:284 10.1186/1471-2458-6-284 ; PubMed Central PMCID: PMCPMC1660563. 17116252 PMC1660563

[15] Kiarie JN, Farquhar C, Richardson BA, Kabura MN, John FN, Nduati RW, et al Domestic violence and prevention of mother-to-child transmission of HIV-1. AIDS. 2006;20(13):1763–9. 10.1097/01.aids.0000242823.51754.0c ; PubMed Central PMCID: PMCPMC3384736. 16931941 PMC3384736

[16] Kohler PK, Ondenge K, Mills LA, Okanda J, Kinuthia J, Olilo G, et al Shame, guilt, and stress: Community perceptions of barriers to engaging in prevention of mother to child transmission (PMTCT) programs in western Kenya. AIDS Patient Care STDS. 2014;28(12):643–51. 10.1089/apc.2014.0171 ; PubMed Central PMCID: PMCPMC4250952. 25361205 PMC4250952

[17] Lichtenstein B. Domestic violence in barriers to health care for HIV-positive women. AIDS Patient Care & STDs. 2006;20(2):122–32. 16475893 10.1089/apc.2006.20.122

[18] Gonzalez JS, Batchelder AW, Psaros C, Safren SA. Depression and HIV/AIDS treatment nonadherence: a review and meta-analysis. Journal of Acquired Immune Deficiency Syndromes. 2011;58(2):181–7. 10.1097/QAI.0b013e31822d490a ; PubMed Central PMCID: PMCPMC3858003. 21857529 PMC3858003

[19] Ramirez-Avila L, Regan S, Giddy J, Chetty S, Ross D, Katz JN, et al Depressive symptoms and their impact on health-seeking behaviors in newly-diagnosed HIV-infected patients in Durban, South Africa. AIDS and Behavior. 2012;16(8):2226–35. 10.1007/s10461-012-0160-y 22451351 PMC3521619

[20] Yotebieng M, Thirumurthy H, Moracco KE, Kawende B, Chalachala JL, Wenzi LK, et al Conditional cash transfers and uptake of and retention in prevention of mother-to-child HIV transmission care: a randomised controlled trial. The Lancet HIV. 2016;3(2):e85–e93. 10.1016/S2352-3018(15)00247-7 26847230 PMC5485848

[21] Yotebieng M, Thirumurthy H, Moracco KE, Edmonds A, Tabala M, Kawende B, et al Conditional cash transfers to increase retention in PMTCT care, antiretroviral adherence, and postpartum virological suppression: a randomized controlled trial. Journal of Acquired Immune Deficiency Syndromes 2016;72:S124–9. 10.1097/QAI.0000000000001062 27355499 PMC5113245

[22] Garcia-Moreno C, Jansen HA, Ellsberg M, Heise L, Watts CH. Prevalence of intimate partner violence: findings from the WHO multi-country study on women's health and domestic violence. The Lancet. 2006;368(9543):1260–9. 10.1016/S0140-6736(06)69523-8 17027732

[23] Ludermir AB, Lewis G, Valongueiro SA, de Araújo TVB, Araya R. Violence against women by their intimate partner during pregnancy and postnatal depression: a prospective cohort study. The Lancet. 2010;376(9744):903–10. 10.1016/S0140-6736(10)60887-2 20822809

[24] McFarlane J, Parker B, Soeken K, Bullock L. Assessing for abuse during pregnancy: severity and frequency of injuries and associated entry into prenatal care. JAMA. 1992;267(23):3176–8. 1593739 10.1001/jama.267.23.3176

[25] Centers for Disease Control and Prevention. Youth Risk Behavior Survey www.cdc.gov/yrbs2007 [cited 2018 August 5].

[26] Rurangirwa AA, Mogren I, Ntaganira J, Krantz G. Intimate partner violence among pregnant women in Rwanda, its associated risk factors and relationship to ANC services attendance: a population-based study. BMJ Open. 2017;7(2):e013155 10.1136/bmjopen-2016-013155 ; PubMed Central PMCID: PMCPMC5337709. 28399509 PMC5337709

[27] Goo L, Harlow SD. Intimate partner violence affects skilled attendance at most recent delivery among women in Kenya. Maternal and Child Health Journal. 2012;16(5):1131–7. 10.1007/s10995-011-0838-1 . 21688110

[28] Mohammed BH, Johnston JM, Harwell JI, Yi H, Tsang KW, Haidar JA. Intimate partner violence and utilization of maternal health care services in Addis Ababa, Ethiopia. BMC Health Services Research. 2017;17(1):178 10.1186/s12913-017-2121-7 ; PubMed Central PMCID: PMCPMC5341201. 28270137 PMC5341201

[29] Rahman M, Nakamura K, Seino K, Kizuki M. Intimate partner violence and use of reproductive health services among married women: evidence from a national Bangladeshi sample. BMC Public Health. 2012;12(913). 10.1186/1471-2458-12-913 PMC3527149 23102051

[30] Israel BA, Rounds KA. Social networks and social support: A synthesis for health educators. Advances in Health Education and Promotion. 1987;2(31):1–35.

[31] Coker AL, Smith PH, Thompson MP, McKeown RE, Bethea L, Davis KE. Social support protects against the negative effects of partner violence on mental health. Journal of women's health & gender-based medicine. 2002;11(5):465–76. 10.1089/15246090260137644 12165164

[32] Nachega JB, Uthman OA, Anderson J, Peltzer K, Wampold S, Cotton MF, et al Adherence to antiretroviral therapy during and after pregnancy in low-income, middle-income, and high-income countries: a systematic review and meta-analysis. AIDS. 2012;26(16):2039–52. 10.1097/QAD.0b013e328359590f ; PubMed Central PMCID: PMCPMC5061936. 22951634 PMC5061936

[33] Tlapek SM. Women's Status and Intimate Partner Violence in the Democratic Republic of Congo. Journal of Interpersonal Violence. 2015;30(14):2526–40. 10.1177/0886260514553118 . 25315479

[34] Alhusen JL, Ray E, Sharps P, Bullock L. Intimate partner violence during pregnancy: maternal and neonatal outcomes. Journal of Women's Health 2015;24(1):100–6. 10.1089/jwh.2014.4872 ; PubMed Central PMCID: PMCPMC4361157. 25265285 PMC4361157

[35] Rodriguez VJ, Peltzer K, Matseke G, Weiss SM, Shine A, Jones DL. Pre-and postnatal exposure to intimate partner violence among South African HIV-infected mothers and infant developmental functioning at 12 months of age. Archives of women's mental health. 2018:1–7. 10.1007/s00737-018-0857-7 29796967

[36] Ministère du Plan et Suivi de la Mise en œuvre de la Révolution de la Modernité (MPSMRM) MdlS, International PMaI. Democratic Republic of Congo Demographic and health survey 2013–14: Key findings. Rockville, Maryland, USA: MPSMRM, MSP et ICF International; 2014.

[37] García-Moreno C, Pallitto C, Devries K, Stöckl H, Watts C, Abrahams N. Global and regional estimates of violence against women: prevalence and health effects of intimate partner violence and non-partner sexual violence: World Health Organization; 2013.

